# Interaction effect of mobile gaming addiction and excessive sugar-sweetened beverage consumption on overweight and obesity among schoolchildren: Evidence from a large population-based study in Guangzhou, China

**DOI:** 10.1556/2006.2024.00086

**Published:** 2025-01-24

**Authors:** Zhengge Jin, Wenxin Ge, Wenwen Bao, Jinghong Liang, Yushan Zhang, Lixin Hu, Yingqi Pu, Meiling Liu, Jiaqi Chen, Xiuzhi Yang, Zhuowen Wu, Yajun Chen

**Affiliations:** Department of Maternal and Child Health, School of Public Health, Sun Yat-sen University, Guangzhou 510080, China

**Keywords:** mobile gaming addiction, internet gaming disorder, sugar-sweetened beverages, overweight/obesity, schoolchildren, interaction

## Abstract

**Objective:**

To explore the individual and interactive associations between mobile gaming addiction (MGA), excessive consumption of sugar-sweetened beverages (SSBs), and overweight/obesity among schoolchildren, and to investigate whether these interactions vary by gender or grade level.

**Methods:**

Data were drawn from the Children's Growth Environment, Lifestyle, and Physical and Mental Health Development project (COHERENCE) conducted in Guangzhou, China, during the 2019/20 academic year. 418,197 children aged 6–12 years were included in the study. All participants were asked to complete an eligible questionnaire to provide details of their MGA over the past three months and SSBs consumption over the past week. Multiplicative and additive interaction models were performed to evaluate the interaction effects of MGA and excessive SSBs consumption on overweight/obesity, and variations by gender and grade level were also examined.

**Results:**

Excessive SSBs consumption was identified as a risk factor for childhood overweight/obesity, but MGA was not. However, the combination of MGA and excessive SSBs consumption was associated with an increased risk of overweight/obesity. This multiplicative interaction was significantly stronger in girls than in boys, with no differences observed across grade levels. Additionally, the additive interaction effect between MGA and excessive SSBs consumption was present only in girls and children in the lower elementary grades.

**Conclusions:**

This cross-sectional study found that the combination of MGA and excessive SSBs consumption is linked to an increased risk of childhood overweight/obesity, particularly in girls and children in lower elementary grades. These findings highlight the importance of addressing these factors together in targeted interventions.

## Introduction

Childhood overweight and obesity are chronic metabolic diseases with rising prevalence worldwide, and recognized as critical preventable public health concerns throughout the lifespan (NCD Risk Factor Collaboration, 2017). A recent study of over 190 countries indicated that obesity affects an estimated 1 billion people globally, with the prevalence among children and adolescents quadrupling from 1990 to 2022 (NCD Risk Factor Collaboration, 2024). In China, 31.8% of school-aged children are predicted to be overweight or obese, potentially resulting in $61 billion in medical costs by 2030 (Wang, Zhao, Gao, Pan, & Xue, 2021). The factors contributing to childhood obesity are multifaceted, encompassing genetic, environmental, nutritional, and behavioral influences (Han, Lawlor, & Kimm, 2010). Among these, lifestyle choices such as excessive consumption of sugar-sweetened beverages (SSBs) and increased screen time, particularly through mobile gaming, have garnered significant attention. Both behaviors independently contribute to energy imbalance—a core determinant of weight gain and obesity (Hall et al., 2011).

Mobile gaming addiction (MGA) is part of internet gaming disorder, which the World Health Organization (WHO) officially classifies as a mental disorder in the International Classification of Diseases, 11th Revision (ICD-11) (Laconi, Pirès, & Chabrol, 2017; Müller et al., 2022). The rise in MGA among children parallels the growing accessibility of smartphones and tablets (China Internet Network Information Center & Central Committee of the Communist Youth League of China, 2023; Sherry, Greenberg, Lucas, & Lachlan, 2006). These devices provide endless entertainment options, often at the expense of physical activity (Kenney & Gortmaker, 2017). Prolonged sedentary behavior, combined with the stimulating and immersive nature of mobile games, significantly reduces overall energy expenditure (Hills, Okely, & Baur, 2010). Concurrently, the consumption of SSBs has become a prevalent dietary habit among children and adolescents in both high-income and low- and middle-income countries (Hu, Song, MacGregor, & He, 2023; Malik & Hu, 2022), providing a substantial source of added sugars and empty calories. Although the “Dietary Guidelines for Chinese Residents (2016)” advised limiting added sugar consumption to no more than 50 g daily, and ideally less than 25 g (Wang, Lay, Yu, & Shen, 2016), it is estimated that 64.8% of school-aged children in China still consume SSBs beyond the recommended levels (Cao, Zhu, Chen, & Jing, 2022). Excessive intake increases total caloric consumption and fails to provide essential nutrients, further contributing to energy imbalance.

MGA and frequent consumption of excessive SSBs have been independently linked to a wide range of adverse health outcomes, including cardiovascular disease, poor sleep quality, type 2 diabetes mellitus, and depressive symptoms (Huang et al., 2023; Jin et al., 2024; Lissak, 2018; Malik & Hu, 2022; Malik, Popkin, Bray, Després, & Hu, 2010). Previous studies have indicated a strong correlation between video games and the consumption of SSBs (Goodman et al., 2020), both of which are independent risk factors for childhood obesity (Kracht, Joseph, & Staiano, 2020; Scharf & DeBoer, 2016). While direct studies on the interaction effects between MGA and SSBs are limited, theoretical frameworks and related evidence suggest possible synergistic effects. MGA may exacerbate the impact of SSBs consumption on Body Mass Index (BMI) through increased sedentary behavior and altered dietary preferences. A recent study by Goodman et al. (2020) has demonstrated a modest but significant association between video game use and increased BMI in later years, with the relationship partly mediated by irregular bedtimes and higher consumption of SSBs. This finding supports the hypothesis that sedentary activities, such as video gaming, coupled with unhealthy dietary practices, can lead to a net positive energy balance, thereby increasing the risk of overweight/obesity. Understanding the synergistic or additive effects of these lifestyle factors is crucial for developing targeted interventions and informing public health strategies.

In the current study, we sought to evaluate the association of MGA and excessive SSBs consumption with childhood overweight/obesity using a large sample of school-age children in Guangzhou, China. We also aimed to investigate whether the interaction between the two conditions is additively associated with an increased risk of childhood overweight/obesity. As the epidemiology of MGA, SSBs, and childhood overweight/obesity is dependent upon gender and age, we examined whether the interaction between MGA and excessive SSBs consumption differed by gender or grade level. We hypothesized that both MGA and excessive SSBs consumption, as well as their interaction, increase the risk of overweight/obesity in Chinese children, and that these associations vary by gender and grade level.

## Methods

### Participants

The current cross-sectional analysis was based on a subset of the children's growth environment, lifestyle, physical, and mental health development project (COHERENCE) (Bao et al., 2024). Details of the project design, including recruitment procedures and selection and exclusion criteria, have been published elsewhere (Bao et al., 2024). Briefly, this is an ongoing cohort study conducted annually across approximately 1,600 primary and middle schools in 11 administrative districts of Guangzhou, situated in southern China. These districts encompass Liwan, Yuexiu, Haizhu, Tianhe, Baiyun, Huadu, Zengcheng, Conghua, Huangpu, Panyu, and Nansha. Since 2016, all students in Guangzhou have been registered in the Electronic Health Records System (EHRS) every year during September and October. This system contains their basic demographic information (such as ID, gender, and birthday), as well as annually updated data from physical examinations and questionnaires. All personal information was strictly processed with desensitization measures.

The study presents a cross-sectional analysis of data obtained from the fourth wave of the COHERENCE, which was conducted in the 2019/20 academic year. A total of 904,470 children aged 6–12 years were selected from the 2020 COHERENCE database. Participants without MGA data (*N* = 1,171) and SSBs data (*N* = 4,459) were excluded. Moreover, 430,239 participants were excluded due to ineligibility for overweight/obesity. Additionally, 50,404 participants were excluded with missing values on variables such as gender, grade level, and other relevant factors. Finally, 418,197 children were included in the statistical analysis.

### Measures

#### Socio-demographic data

Participants' socio-demographic variables and lifestyle factors associated with overweight/obesity were collected, including the child's age (years), gender (boys or girls), grade level [lower elementary grades (grades 1–3) vs. higher elementary grades (grades 4–6)], only child (yes or no), parents' educational level (below senior high school, completed senior high school, completed junior college, completed college or above), paternal smoking status [never smokers (both parents had never smoked), former smokers (either parent was a former smoker), and current smokers (either parent was a current smoker)], maternal smoking status [never smokers (both parents had never smoked), former smokers (either parent was a former smoker), and current smokers (either parent was a current smoker)], household monthly income (<5,000, 5,000–7,999, 8,000–11,999, ≥12,000 CNY, refused to answer), and average outdoor PA time (<2 hour/day, ≥2 hour/day).

#### Mobile gaming addiction

Participants were assessed for MGA over the past three months using the short form of Problematic Mobile Gaming Questionnaire (PMGQ-SF), a valid measure validated by diagnostic criteria, which also provided a cut-off point corresponding to the clinical diagnosis (Pan, Chiu, & Lin, 2019). The PMGQ-SF consists of four items assessing MGA, including: (1) “Due to prolonged mobile gaming, I experience eye strain, muscle pain, or other physical discomfort.”; (2) “I often find myself picking up my phone to play games, even when I originally didn't intend to.”; (3) “In the past three months, I have felt the need to play mobile games more frequently or for longer periods to achieve satisfaction.”; and (4) “I feel restless or irritable if I am unable to play mobile games.” These items evaluate various aspects of MGA, including physical discomfort, impulsive gaming behavior, increased tolerance, and withdrawal symptoms. For students in grades 1 to 3, parents assisted their children in completing the questionnaire to ensure accurate understanding and responses. Students in higher grades (grades 4–6) completed the questionnaire independently. Participants were instructed to rate the items using a 4-point Likert scale (1, strongly disagree; 2, somewhat disagree; 3, somewhat agree; and 4, strongly agree). Respondents scoring 10 or higher were classified as exhibiting MGA. The Cronbach's α of 0.88 indicated that the scale was internally consistent.

#### Excessive sugar-sweetened beverages consumption

The consumption of SSBs by children and their parents was assessed using the following question: “In the last week, how many times did your child usually drink SSBs (such as Coke, Sprite, fruit punch, fruit milk, energy drinks, milk tea, coconut milk, etc.) that contain more than 250 ml?” (Gui et al., 2021). Participants were classified as having excessive SSBs consumption if they reported consuming SSBs ≥6 times/week in the study.

#### Overweight/obesity

Height and weight measurements were conducted using height and weight scales calibrated by the local health department for student physical examinations. Participants were instructed to remove coats, shoes, and hats, and to stand on the measurement platform wearing lightweight clothing with an upright posture, heels together, toes pointing outward, arms naturally hanging down, and eyes facing forward. Subsequently, their height and weight were measured with accuracies of 0.1 cm and 0.1 kg, respectively. All measurements were taken in the participants' fasted state early in the morning. Once the instrument readings stabilized, the readings were taken and recorded by the measuring personnel. Measurements were conducted twice consecutively, with a maximum allowable error of 1.0 cm for height and 0.5 kg for weight, and the average value was calculated. Physical examinations were conducted by trained investigators using standardized methods. All participants underwent measurements of height and weight, from which BMI was calculated as weight (in kilograms) divided by height (in meters) squared (kg/m^2^). Overweight and obesity were defined by the Screening for overweight and obesity among school-age children and adolescents (WST586-2018) (National Health and Family, 2018). Individuals with a BMI greater than or equal to the “overweight” cut-off point for their respective gender and age group, but less than the “obesity” cut-off point, were classified as overweight. Individuals with a BMI greater than or equal to the “obesity” cut-off point for their respective gender and age group were classified as obese.

### Statistical analysis

Data were described as means and standard deviations (SDs) for continuous variables, and frequency (*n*) with percentage (%) was used to describe categorical variables. Baseline characteristics were compared between groups using *t*-tests and chi-square (*χ*^2^) tests where appropriate. Binomial and multinomial logistic regression analyses were performed to obtain the odds ratios (*ORs*) and 95% confidence intervals (*CIs*) for the associations among GAM, excessive SSBs consumption and overweight/obesity in Chinese schoolchildren.

To quantify the additive and multiplicative interactions, we additionally included a product term of GAM and excessive SSBs consumption in the binomial logistic regression model. The *OR* with its 95%*CI* of the product term was the measure of interaction on the multiplicative scale. Gender and grade level differences in the associations were examined by two odds ratios (Ratio of two odds ratios, *RORs*) (Altman & Bland, 2003). The additive interactive effects of GAM and excessive SSBs consumption on overweight/obesity were assessed using three distinct metrics: relative excess risk of interaction (RERI), attributable proportions (AP), and synergy index (SI) (Andersson, Alfredsson, Källberg, Zdravkovic, & Ahlbom, 2005). These metrics comprehensively capture various facets of interaction, encompassing the portion of the effect directly attributable to interaction, the proportion of the combined effect stemming from interaction, and the ratio between the combined effect and individual effects. Specifically, RERI and AP were not equal to 0 and SI was not equal to 1 indicating interaction developed (the combined effect of GAM and excessive SSBs consumption was not equal to the sum of their individual effects) (Li & Chambless, 2007). All analyses were performed using R, version 3.2.3 (R Group for Statistical Computing). All tests of significance were 2-sided, and *p* < 0.05 was considered statistically significant.

### Ethics

All study procedures and protocols were reviewed and approved by the Human Studies Committee of Sun Yat-sen University (approval number: L2016-010). Informed written consent was obtained from all participating students and their parents or guardians. The study adhered to the Strengthening the Reporting of Observational Studies in Epidemiology (STROBE) reporting guidelines.

## Results

### Sample characteristics

Among the 418,197 participants, the mean age was 9.55 years (SD 1.4), with 54.9% boys and 43.6% of the students in the lower elementary grades (Table 1). Results revealed that the prevalence of MGA was 21.1%, and approximately 1.4% (*n* = 6,023) of the participants reported excessive consumption of SSBs (Table 1). Overall, 74,325 (17.8%) students were identified as overweight/obese. Overweight/obesity were more common in boys, students in the higher elementary grades, and those who were the only child in the family. Additionally, children with higher-educated parents, smoking habits, higher household incomes, and more than two hours of average daily outdoor PA showed higher prevalence of overweight/obesity (*p* < 0.001). Furthermore, children with MGA and excessive SSBs consumption also showed increased morbidity of overweight/obesity (*p* < 0.001).

**Table 1.** Comparison of the prevalence of overweight/obesity in participants with different characteristics (*n* = 418,197)

Variable	Total	Overweight/obesity	*P*-value^a^
Age y [mean (SD)]	9.55 (1.4)	9.69 (1.4)	<0.001
Gender [*n* (%)]			
Boys	229,769 (54.9)	50,820 (22.1)	<0.001
Girls	188,428 (45.1)	23,505 (12.5)	
Grade level [*n* (%)]			
Lower elementary grades (Grades 1–3)	182,374 (43.6)	28,876 (15.8)	<0.001
Higher elementary grades (Grades 4–6)	235,823 (56.4)	45,449 (19.3)	
Only child [*n* (%)]			
Yes	100,833 (24.1)	20,442 (20.3)	<0.001
No	317,364 (75.9)	53,883 (17.0)	
Paternal education level [*n* (%)]			
Below senior high school	143,224 (34.2)	24,021 (16.8)	<0.001
Completed senior high school	114,727 (27.4)	20,211 (17.6)	
Completed junior college	74,006 (17.7)	13,266 (17.9)	
Completed college or above	86,240 (20.6)	16,827 (19.5)	
Maternal education level [*n* (%)]			
Below senior high school	154,296 (36.9)	26,314 (17.1)	<0.001
Completed senior high school	105,253 (25.2)	18,657 (17.7)	
Completed junior college	83,747 (20.0)	14,858 (17.7)	
Completed college or above	74,901 (17.9)	14,496 (19.4)	
Paternal smoking status [*n* (%)]			
Never smokers	189,135 (45.2)	32,757 (17.3)	<0.001
Former smokers	46,796 (11.2)	8,433 (18.0)	
Current smokers	182,266 (43.6)	33,135 (18.2)	
Maternal smoking status [*n* (%)]			
Never smokers	413,968 (99.0)	73,452 (17.7)	<0.001
Former smokers	2,132 (0.5)	431 (20.2)	
Current smokers	2,097 (0.5)	442 (21.1)	
Household monthly income [*n* (%)]			
<5,000 CNY	171,854 (41.1)	27,681 (16.1)	<0.001
5,000–7,999 CNY	86,553 (20.7)	15,506 (17.9)	
8,000–11,999 CNY	51,389 (12.3)	9,753 (19.0)	
≥12,000 CNY	66,513 (15.9)	13,921 (20.9)	
Refused to answer	41,888 (10.0)	7,464 (17.8)	
Average outdoor PA time [*n* (%)]			
<2 (hour/day)	361,140 (86.4)	63,767 (17.7)	<0.001
≥2 (hour/day)	57,057 (13.6)	10,558 (18.5)	
MGA {score [*n* (%)]}			
No (<10)	330,069 (78.9)	58,219 (17.6)	<0.001
Yes (≥10)	88,128 (21.1)	16,106 (18.3)	
Excessive SSBs [*n* (%)]			
No (<6 times/week)	412,174 (98.6)	73,057 (17.7)	<0.001
Yes (≥6 times/week)	6,023 (1.4)	1,268 (21.1)	

*Note*. Dependent continuous variables were presented as mean (SD), and categorical variables were presented as *n* (%).

^a^ The *p*-values were assessed by two independent samples *t*-test (continuous variables) or by chi-square test (categorical variables) to represent the childhood overweight/obesity disparities.

*Abbreviations*: SD, standard deviation; CNY, China Yuan; PA, physical activity; MGA, mobile gaming addiction; SSBs, sugar-sweetened beverages.

### Association of MGA, excessive SSBs consumption and overweight/obesity in Chinese children

Table 2 demonstrated the independent associations of MGA and excessive consumption of SSBs with overweight/obesity in Chinese children. Multivariate logistic regression analysis indicated that excessive SSBs consumption (*OR* = 1.14, 95%*CI*: 1.07–1.22) were risk factors for overweight/obesity in the total sample compared with the control group, whereas MGA was not statistically associated with overweight/obesity. After adjusting for confounding factors, gender-stratified analyses revealed that excessive consumption of SSBs was associated with a higher risk of overweight/obesity for both boys (*OR* = 1.13, 95%*CI*: 1.04–1.21) and girls (*OR* = 1.17, 95%*CI*: 1.04–1.31). In contrast, MGA did not show a significant association with an increased risk of overweight/obesity. Meanwhile, similar results were found in the grade-level stratified analyses. No gender- or grade-differences were observed in the independent effects of MGA or SSBs on overweight/obesity in children.

**Table 2.** Associations of MGA, excessive SSBs consumption and overweight/obesity in Chinese schoolchildren

Groups	MGA		Excessive SSBs	
	No	Yes	No	Yes
Total				
*AOR* (95%*CI*)^a^	1.00	1.00 (0.98–1.02)	1.00	1.14 (1.07–1.22)***
Boys				
*AOR* (95%*CI*)^b^	1.00	0.99 (0.97–1.01)	1.00	1.13 (1.04–1.21)*
Girls				
*AOR* (95%*CI*)^b^	1.00	1.02 (0.98–1.05)	1.00	1.17 (1.04–1.31)*
*ROR* (95%*CI*)^d^		0.97 (0.93–1.01)		0.97 (0.84–1.11)
Lower elementary grades (Grades 1–3)				
*AOR* (95%*CI*)^c^	1.00	1.01 (0.98–1.04)	1.00	1.19 (1.06–1.33)*
Higher elementary grades (Grades 4–6)				
*AOR* (95%*CI*)^c^	1.00	0.99 (0.97–1.02)	1.00	1.12 (1.04–1.21)*
*ROR* (95%*CI*)^e^		1.02 (0.98–1.06)		1.06 (0.93–1.22)

*Note*. ^a^Adjusted for age, gender, grade level, only child, parents' education level, paternal smoking status, maternal smoking status, household monthly income (CNY), average outdoor PA time.

^b^Adjusted for age, grade level, only child, parents' education level, parents' smoking status, household monthly income (CNY), average outdoor PA time.

^c^Adjusted for age, gender, only child, parents' education level, parents' smoking status, household monthly income (CNY), average outdoor PA time.

^d^Gender differences in the associations were examined via two odds ratios (*RORs*).

^e^Grade level differences in the associations were examined via two odds ratios (*RORs*).

*p-value <0.05, **p-value <0.01, ***p-value <0.001.

*Abbreviations*: MGA, mobile gaming addiction; SSBs, sugar-sweetened beverages; *AOR*, adjusted odd ratio; *CI*, confidence interval; PA, physical activity; *RORs*, ratio of two odds ratios; CNY, China Yuan.

### Multiplicative interactions of MGA, excessive SSBs consumption and overweight/obesity in Chinese children

As indicated in Table A1 (Appendix Table A1), MGA and excessive consumption of SSBs were highly associated. The results of the regression analysis examining the multiplicative interactions between MGA and excessive consumption of SSBs on overweight/obesity were presented in Table 3. Compared to the reference group (no MGA and no excessive SSBs consumption), a significant multiplicative interaction effect of MGA and excessive consumption of SSBs on overweight/obesity was observed in all groups—total sample, boys, girls, students in the lower elementary grades, and students in the higher elementary grades—after adjusting for confounding factors. Specifically, when MGA was excessive, the risk of overweight/obesity in children with excessive consumption of SSBs increased. The multiplicative interaction of MGA and excessive SSBs consumption on overweight/obesity was significantly stronger in girls compared to boys (MGA × excessive SSBs consumption: *ROR* = 0.78, 95%*CI*: 0.61–1.00), as evidenced in Table 3. However, no additional variations were observed across different grade groups regarding the multiplicative interaction of MGA and excessive SSBs consumption on overweight/obesity.

**Table 3.** Multiplication interactions of MGA, excessive SSBs consumption and overweight/obesity in Chinese schoolchildren

Groups	MGA × Excessive SSBs	
	No × No	Yes × Yes
Total		
*AOR* (95%*CI*)^a^	1.00	1.26 (1.12–1.41)***
Boys		
*AOR* (95%*CI*)^b^	1.00	1.16 (1.02–1.33)*
Girls		
*AOR* (95%*CI*)^b^	1.00	1.49 (1.21–1.84)***
*ROR* (95%*CI*)^d^		0.78 (0.61–1.00)*
Lower elementary grades (Grades 1–3)		
*AOR* (95%*CI*)^c^	1.00	1.45 (1.17–1.80)***
Higher elementary grades (Grades 4–6)		
*AOR* (95%*CI*)^c^	1.00	1.18 (1.03–1.35)*
*ROR* (95%*CI*)^e^		1.23 (0.95–1.58)

*Note*. Categorical variables were presented as *n* (%).

^a^Adjusted for age, gender, grade level, only child, parents' education level, paternal smoking status, maternal smoking status, household monthly income (CNY), average outdoor PA time.

^b^Adjusted for age, grade level, only child, parents' education level, parents' smoking status, household monthly income (CNY), average outdoor PA time.

^c^Adjusted for age, gender, only child, parents' education level, parents' smoking status, household monthly income (CNY), average outdoor PA time.

^d^Gender differences in the associations were examined via two odds ratios (*RORs*).

^e^Grade level differences in the associations were examined via two odds ratios (*RORs*).

*p-value <0.05, **p-value <0.01, ***p-value <0.001.

*Abbreviations*: MGA, mobile gaming addiction; SSBs, sugar-sweetened beverages; *AOR*, adjusted odd ratio; *CI*, confidence interval; PA, physical activity; *RORs*, ratio of two odds ratios; CNY, China Yuan.

### Additive interactions of MGA, excessive SSBs consumption and overweight/obesity in Chinese children

Table 4 showed the additive interaction effects of MGA and excessive consumption of SSBs among Chinese children (MGA × excessive SSBs consumption: RERI = 0.17 [0.01–0.32], AP = 0.13 [0.02–0.24], SI = 2.79 [1.13–6.88]). Specifically, when MGA and excessive SSBs consumption coexisted, the risk of overweight/obesity in children increased. An additive interaction effect between MGA and excessive consumption of SSBs on overweight/obesity was observed in girls (MGA × excessive SSBs consumption: RERI = 0.42 [0.07–0.78], AP = 0.28 [0.10–0.47], SI = 6.97 [0.79–61.32]) and students in the lower elementary grades (MGA × excessive SSBs consumption: RERI = 0.35 [0.01–0.70], AP = 0.24 [0.05–0.44], SI = 4.48 [0.86–23.32]). However, this interaction was not significant in boys and students in the higher elementary grades.

**Table 4.** Additive interactions of MGA, excessive SSBs consumption and overweight/obesity in Chinese schoolchildren

	Model	*β*	*AOR* (95% *CI*)	RERI	AP	SI
Total^a^						
MGA × Excessive SSBs	Yes × Yes	0.229	1.26 (1.12–1.41)***	0.17 (0.01–0.32)	0.13 (0.02–0.24)	2.79 (1.13–6.88)
	No × Yes	0.092	1.10 (1.02–1.18)*			
	Yes × No	−0.004	1.00 (0.98–1.02)			
	No × No		1.00			
Boys^b^						
MGA × Excessive SSBs	Yes × Yes	0.150	1.16 (1.02–1.33)*	0.07 (−0.12–0.26)	0.06 (−0.10–0.22)	1.75 (0.41–7.45)
	No × Yes	0.100	1.11 (1.01–1.21)*			
	Yes × No	−0.013	0.99 (0.96–1.01)			
	No × No		1.00			
Girls^b^						
MGA × Excessive SSBs	Yes × Yes	0.401	1.49 (1.21–1.85)***	0.42 (0.07–0.78)	0.28 (0.10–0.47)	6.97 (0.79–61.32)
	No × Yes	0.058	1.06 (0.92–1.22)			
	Yes × No	0.011	1.01 (0.98–1.05)			
	No × No		1.00			
Lower elementary grades (Grades 1–3)^c^						
MGA × Excessive SSBs	Yes × Yes	0.375	1.46 (1.18–1.80)***	0.35 (0.01–0.70)	0.24 (0.05–0.44)	4.48 (0.86–23.32)
	No × Yes	0.093	1.10 (0.95–1.26)			
	Yes × No	0.004	1.00 (0.97–1.04)			
	No × No		1.00			
Higher elementary grades (Grades 4–6)^c^						
MGA × Excessive SSBs	Yes × Yes	0.160	1.17 (1.03–1.35)	0.09 (−0.10–0.28)	0.08 (−0.07–0.23)	2.17 (0.48–9.92)
	No × Yes	0.087	1.09 (1.00–1.19)			
	Yes × No	−0.011	0.99 (0.96–1.01)			
	No × No		1.00			

*Note*. ^a^Adjusted for age, gender, grade level, only child, parents' education level, paternal smoking status, maternal smoking status, household monthly income (CNY), average outdoor PA time.

^b^Adjusted for age, grade level, only child, parents' education level, parents' smoking status, household monthly income (CNY), average outdoor PA time.

^c^Adjusted for age, gender, only child, parents' education level, parents' smoking status, household monthly income (CNY), average outdoor PA time.

*p-value <0.05, **p-value <0.01, ***p-value <0.001.

*Abbreviations*: MGA, mobile gaming addiction; SSBs, sugar-sweetened beverages; *AOR*, adjusted odd ratio; *CI*, confidence interval; PA, physical activity; *RORs*, ratio of two odds ratios; CNY, China Yuan; RERI, relative excess risk due to interaction; AP, attributable proportion of interaction; SI, synergy index.

## Discussion

In this large population-based study of 418,197 primary schoolchildren, we found that excessive consumption of SSBs was associated with an increased risk of overweight/obesity, but MGA was not. Moreover, interaction analysis revealed that MGA and excessive SSBs consumption had both multiplicative and additive interactive impacts on the overweight/obesity of children, with similar associations observed across gender and grade groups. Yet the multiplicative interaction of MGA and excessive SSBs consumption on overweight/obesity was significantly stronger in girls than in boys, with no additional variations observed across different grade groups. Meanwhile, the additive interaction effect between MGA and excessive SSBs consumption on overweight/obesity was detected only in girls and children in the lower elementary grades.

The prevalence rate of 17.8% for overweight/obese children aged 6–12 years in this survey was lower than that reported for European children of the same age in previous literatures (Ogden, Carroll, Kit, & Flegal, 2012; WHO, 2023), but higher than the overall rate for Chinese children (Hong, Ullah, Wang, & Fu, 2023; X. F. Pan, Wang, & Pan, 2021). This discrepancy might be attributed to differences in the criteria used for defining overweight and obesity among children. Our study utilized the guidelines from the Chinese National Standard (WS/T586-2018), which establish specific BMI cutoff points for overweight and obesity based on gender and age groups, aiming to better reflect the growth patterns of Chinese children (National Health and Family, 2018). In contrast, European studies often employed the WHO growth standards, whose universal BMI thresholds might not accurately capture the unique growth patterns of children in diverse regions (WHO, 2007). Furthermore, factors such as dietary habits, lifestyle, socioeconomic conditions, and environmental influences might also contribute to these variations in prevalence rates (Ruiz, Zuelch, Dimitratos, & Scherr, 2019). It was observed that overweight/obesity was more likely to occur in boys and higher-grade children, which was consistent with findings from other studies (Hong et al., 2023; Rogers et al., 2023; Zhang et al., 2021). A higher prevalence of childhood overweight/obesity was more likely in only child than in non-only child (Min, Xue, Wang, Li, & Wang, 2017). Similarly, we observed that children with a higher parental education level, smoking habits, higher household incomes were more likely to display a higher prevalence of overweight/obesity than control subjects (Gao, Wells, Johnson, & Li, 2022; Lecorguillé et al., 2023; Muthuri et al., 2016). Contrary to the findings from Stone et al. study (Stone & Faulkner, 2014), children who engaged in ≥2 h of average daily outdoor PA were more likely to report being overweight or obese, which might be partly attributed to the fact that most of their exercise is of low intensity and insufficient to offset the calories consumed through their diet. Additionally, overweight children might participate in more PA to help manage their weight.

The results showed that MGA was present in about 21.1% of the children, but this was lower than what Hou et al. found (Hou et al., 2022), probably due to scale differences, which used the short form of the Internet Gaming Disorders Scale (IGDS9-SF) (Hou et al., 2022). Most research on game addiction and obesity has focused on internet gaming addiction (Ko, Lin, Lin, & Yen, 2020; Kracht et al., 2020), with very little research addressing MGA (Ma, Wang, Li, & Jia, 2021). The expected positive association of MGA and childhood overweight/obesity was not observed in our study. The results might have been influenced by certain methodological limitations, particularly unaddressed confounding factors such as eating attitudes, sleep patterns, urbanization, and other relevant variables.

The prevalence of the habit of excessive SSBs consumption was lower than what has been observed in other Chinese study (Gan et al., 2021), which could be attributable to regional differences and the impact of COVID-19 on consumer behavior. Specifically, the COVID-19 pandemic has precipitated a decline in the production, transportation, and distribution networks for SSBs, thereby diminishing accessibility for children (Jiang et al., 2023). Our findings of the positive association of excessive SSBs consumption with increased risk of overweight and obesity were also supported by large national cross-sectional studies, prospective cohort studies and randomized clinical trials in children (Hu et al., 2023; Malik, Pan, Willett, & Hu, 2013; Nguyen et al., 2023). The potential mechanisms underlying the relationship have been proposed by previous studies. One explanation is that consuming high-sugar drinks can have long-term effects on the brain's reward circuits, potentially leading to altered food preferences and addictive behaviours (Edwin Thanarajah et al., 2023). Additionally, consumption of beverages contained high levels of added sugar, decreases satiety, leading to incomplete energy compensation at subsequent meals and contributing to weight gain (Malik et al., 2010).

In this study, positive multiplicative and additive interactions between MGA and excessive intake of SSBs on childhood overweight/obesity were observed. Specifically, the additive interaction between MGA and excessive SSBs consumption increased the probability of overweight/obesity in children by 0.17 times. Additionally, 13% of the overweight/obesity cases in children were attributed to this additive interaction. Children with MGA and high consumption of SSBs were 2.79 times more likely to be overweight or obese compared to the sum of the two individual effects in the additive model. Focusing only on results from the multiplicative scale may lead to the misidentification of the high-risk population. Hence, assessing additive interactions is considered most appropriate for public health decision-making, as it better guides the development of disease prevention, control measures, and interventions (Knol & VanderWeele, 2012). The potential synergism may be explained in part by a common pathway in the pathogenesis of childhood overweight/obesity, in which the brain's reward system plays a key role. Both gaming and SSBs consumption activate the brain's reward system to release dopamine, a neurotransmitter associated with pleasure. This simultaneous activation can create an enhanced reward response, leading to behaviors that are more likely to result in obesity than either factor alone (Edwin Thanarajah et al., 2023; Lissak, 2018). Evidence regarding the synergistic effects of MGA and excessive SSBs consumption on childhood overweight/obesity is scarce, and further studies are required to confirm this finding and elucidate the specific mechanisms underlying these synergistic effects.

Furthermore, gender-stratified analysis showed that the multiplicative and additive interactions of MGA and excessive SSBs consumption on childhood overweight/obesity were more significant in girls. This may be due to differences in physiological and behavioural patterns of girls, such as higher body fat percentage and lower physical activity (Guthold, Stevens, Riley, & Bull, 2020; Shah, Tombeau Cost, Fuller, Birken, & Anderson, 2020; Zhao et al., 2023). There were no grade-level differences in the multiplicative interaction effects of MGA and excessive SSBs consumption on overweight/obesity, but the additive interaction effects were significantly stronger in lower-grade students than in higher-grade students. One possible explanation is that younger children, who have weaker self-control, are more likely to make unhealthy behavioral choices (Baker, Morawska, & Mitchell, 2019; Ha et al., 2016), thereby increasing the risk of overweight and obesity. These findings highlight the necessity for public health policies and interventions to prioritize early childhood development, particularly targeting girls and younger students, to mitigate the impact of MGA and SSBs consumption on overweight and obesity. The effectiveness of these strategies should be further investigated in subsequent studies.

### Strengths and limitations

Our research possessed several strengths. The large sample size allowed robust sub-group analyses with sufficient statistical power. Simultaneously, the present study not only estimated the individual effects of GMA or excessive SSBs consumption on overweight/obesity, but also implemented a recently developed epidemiological assessment tool to test their interaction effects on both the additive and multiplicative scales.

The study presents several limitations that should be acknowledged. First, there is limited ability to establish causality given the observational cross-sectional design. This design cannot determine the directionality of relationships, meaning that while associations can be observed, causal conclusions are not possible. Nevertheless, the hypotheses proposed in this study maintain a strong intuitive appeal. Second, the self-reported status of MGA and SSBs consumption may be subject to recall bias. To mitigate this, future research should consider incorporating more objective measurement tools, such as electronic data logs or tracking applications, to validate self-reported data. Third, intriguingly, excessive intake of SSBs during MGA was associated with an increased risk of overweight/obesity in children, but the reasons for this are not fully clarified. Unfortunately, we did not have detailed information regarding dietary intake to calculate the consumption of highly refined sugars and the timing of consumption that may have contributed to our understanding of this linkage. Additionally, the selected threshold for SSBs intake did not fully account for variations in sugar and caloric content among different beverages, potentially affecting the precision of our results, especially in individual-level assessments. In future studies, comprehensive and objective measurement methods, such as dietary records and biomarker analysis, should be considered to assess SSBs consumption better and adjust the threshold based on more precise measures of sugar and caloric intake, thereby enhancing the accuracy and comparability of the research. Finally, while data were collected during a period when the COVID-19 pandemic was effectively controlled in our country, with target areas and schools resuming normal operations, the potential influence of the pandemic or other confounding factors cannot be entirely excluded. Our study included children only from Guangzhou, China, and caution should be exercised when generalizing the findings to other population groups. Future prospective cohort studies should be conducted to provide more robust and generalizable findings from diverse and representative regional samples.

### Implications

Our finding underscores the importance of developing comprehensive public health interventions that address multiple lifestyle factors simultaneously. First, schools should incorporate education on healthy eating and media use into their curricula, teaching students to recognize the dangers of SSBs and MGA, along with practical strategies for moderation and healthier alternatives (e.g., natural juices and engage in more outdoor activities) (Verduci et al., 2021). These programs should be tailored to the age groups of students to ensure appropriateness and effectiveness of the information. Second, the significant impact on girls and younger students highlights the need for targeted approaches. Gender-specific programs that consider the different engagement patterns of boys and girls can enhance the effectiveness of interventions. Moreover, initiatives targeting younger children should involve parents and caregivers to ensure a supportive environment at home, reinforcing healthy behaviors. It has been suggested that family rules can play a crucial role in helping children modify their dietary and sedentary habits, which are significant modifiable risk factors for childhood obesity (Lederer, King, Sovinski, & Kim, 2015). Third, policymakers can leverage these findings to inform and develop regulations that address these dual risk factors. This could include stricter marketing restrictions on sugary beverages aimed at children and guidelines for limiting screen time in educational settings. Several countries and regions globally have implemented taxes on SSBs (Malik & Hu, 2022), but it has not received much attention in our country yet. Future research should continue to explore these interactions and potential mechanisms and develop targeted interventions to support healthier lifestyles among children.

## Conclusions

The study contributes to a better understanding of the independent and combined effects of MGA and excessive SSBs consumption on the risk of overweight/obesity. To the best of our knowledge, this is the first study to examine the coexistence effect of MGA and excessive SSBs consumption on childhood overweight/obesity. We found a positive additive interaction between MGA and excessive SSBs consumption, increasing the risk of overweight and obesity, with this interaction being significant only in girls and students in the lower elementary grades. Longitudinal studies should explore the factors responsible for this interaction and investigate the underlying mechanisms to guide obesity-targeted intervention efforts.

## Data Availability

The survey is not publicly available and participants were protected under a certificate of confidentiality issued by the Government of Guangzhou due to the sensitivity nature of data collected from all students group in Guangzhou city. Requests to assess the dataset from qualified researchers trained in human participant confidentiality protocols may be sent to the School of public Health, Medical College of Sun Yat-Sen University at chenyj68@mail.sysu.edu.cn.

## Appendix

**Table A1.** The prevalence of excessive SBBs consumption by MGA (*n* = 418,197)

Variable	Excessive SBBs		*χ*^2^ value
	No [*n* (%)]	Yes [*n* (%)]	
Total			
MGA			181.011***
No	325,738 (98.7)	4,331 (1.3)	
Yes	86,436 (98.1)	1,692 (1.9)	
Boys			
MGA			99.217***
No	174,885 (98.5)	2,619 (1.5)	
Yes	51,167 (97.9)	1,098 (2.1)	
Girls			
MGA			68.538***
No	150,853 (98.9)	1,712 (1.1)	
Yes	35,269 (98.3)	5,94 (1.7)	
Lower elementary grades (Grades 1–3)			
MGA			52.214***
No	144,916 (99.0)	1,405 (1.0)	
Yes	35,551 (98.6)	502 (1.4)	
Higher elementary grades (Grades 4–6)			
MGA			113.553***
No	180,822 (97.7)	2,926 (1.6)	
Yes	50,885 (97.7)	1,190 (2.3)	

*Note*. Statistical methods: chi-square test.

**p* < 0.05, ***p* < 0.01, ****p* < 0.001.

*Abbreviations*: SSBs, sugar-sweetened beverages; MGA, mobile gaming addiction.
